# Does Soil Nutrient Heterogeneity Improve the Growth Performance and Intraspecific Competition of the Invasive Plant *Myriophyllum aquaticum*?

**DOI:** 10.3389/fpls.2019.00723

**Published:** 2019-05-30

**Authors:** Nan Shen, Hongwei Yu, Siqi Yu, Dan Yu, Chunhua Liu

**Affiliations:** ^1^The National Field Station of Freshwater Ecosystem of Liangzi Lake, Department of Ecology, College of Life Sciences, Wuhan University, Wuhan, China; ^2^Center for Water and Ecology, State Key Joint Laboratory of Environment Simulation and Pollution Control, School of Environment, Tsinghua University, Beijing, China

**Keywords:** soil nutrient heterogeneity, competition, invasive, aquatic plants, *Myriophyllum aquaticum*

## Abstract

Spatial heterogeneity in soil nutrient availability is considered to play an important role in promoting plant invasion success and can affect interspecific competition. Although some clonal plants have been demonstrated to be correlated with resource heterogeneity in terrestrial systems, little is known about how soil nutrient heterogeneity affects the growth of invasive aquatic plants or their population structure. A greenhouse experiment was therefore conducted to study the response of the invasive aquatic plant *Myriophyllum aquaticum* to the spatial heterogeneity of soil nutrients under three plant densities (one, four, or twelve plants 0.28 m^2^) with a constant amount of soil nutrients. The results showed that soil nutrient heterogeneity significantly increased the number of shoots in the single-plant density treatment. However, heterogeneous soil nutrient treatment significantly increased the number of shoots at the expense of total biomass and aboveground biomass in the twelve-plant density treatment. The heterogeneous soil nutrient treatment had low effects on other growth traits and intraspecific competition under different plant density treatments. These results indicate that spatial heterogeneity in soil nutrient availability may facilitate the spread of *M. aquaticum*.

## Introduction

Spatial soil heterogeneity is common in natural habitats (Farley and Fitter, 1999; James et al., 2010) and has positive effects on the performance of clonal plants (Day et al., 2003a; Wacker et al., 2008; Zhou et al., 2012; You et al., 2014). Clonal plants respond to resource heterogeneity by concentrating more nutrient-absorbing organs (e.g., roots or ramets) where nutrient levels are relatively high (Wijesinghe et al., 2001; Hodge, 2004; Kroon et al., 2010; Gao et al., 2012). Some studies have shown that soil nutrient heterogeneity increases the growth performance of individual plants or plant populations (Wacker et al., 2008; Zhou et al., 2012; Xue et al., 2016). For example, plant yield can be enhanced through physiological integration in heterogeneous conditions (Wijesinghe and Hutchings, 1997; Hutchings and Wijesinghe, 2008). However, soil nutrient heterogeneity does not always play a positive role in plant growth, and any positive effect may be eliminated if resources become limited (Roiloa and Retuerto, 2006; Zhang and He, 2009; Dong et al., 2015).

Intraspecific competition can affect the growth performance and reproductive values of plant individuals in a population and, as a consequence, the effective structure and size of the population (Heywood, 1986; Hartl and Clark, 1989; Kleunen et al., 2001). For example, intraspecific competition significantly affects the number and size of ramets of *Ranunculus reptans* (Kleunen et al., 2001) and significantly affects the stolon length and dry mass of *Alternanthera philoxeroides* (Zhou et al., 2012).

Soil nutrient heterogeneity can affect the competitive relationship between plants (Ritchie and Olff, 1999; Hutchings et al., 2003; Van et al., 2011; Mommer et al., 2012). The relationship between the degree of nutrient heterogeneity and the intensity of competition changes dynamically (Kume et al., 2006; Maestre and Reynolds, 2007; Van et al., 2011). For example, soil nutrient heterogeneity can increase the intensity of competition (Fransen et al., 2001; Day et al., 2003b) because the nutrient-absorbing organs of neighboring plants would be concentrated in a smaller area of high quality in heterogeneous environments. However, Zhou et al. (2012) and Yu et al. (2018) found that soil nutrient heterogeneity does not increase competition when plants are genetically identical. Another theory is that a significant effect of soil heterogeneity on intraspecific or interspecific competition may be caused by the differences between plants in their ability to concentrate their nutrient-absorbing organs where resource levels are high (Fransen et al., 2001; Bliss et al., 2002; Dong et al., 2015).

Many alien invasive plants have the capacity for clonal growth (Liu et al., 2006). However, the understanding of the responses of invasive clonal plants to soil nutrient heterogeneity remains limited. Thus, to investigate how soil nutrient heterogeneity affects the growth performance and intraspecific competition of invasive clonal plants, we conducted an experiment with the stoloniferous, invasive clonal plant *Myriophyllum aquaticum*. We hypothesized that soil nutrient heterogeneity will significantly increase the growth performance and influence the intraspecific competition intensity of *M. aquaticum*.

## Materials and Methods

### The Species

*Myriophyllum aquaticum* (Vell.) Verdc., a perennial aquatic clonal plant is widely distributed in tropical and temperate regions such as South America (Sutton, 1985). This species can grow in a broad range of habitats, from semi-moist to semi-submersed conditions (Hussner et al., 2010) and forms dense floating mats by producing creeping stolons over the water surface (Wersal and Madsen, 2011). *M. aquaticum* produces flowers and fruits from April to September (Sutton, 1985). It is increasingly becoming a harmful weed in shallow streams and shallow lakes of North America (Sutton, 1985; Wersal and Madsen, 2011). The *M. aquaticum* plants used in this experiment were collected from natural plant populations at the National Field Station of Freshwater Ecosystem of Liangzi Lake (N 30°05′–30°18′, E 114°21′–114°39′) in Hubei Province, China. All of the collected plant materials were planted in containers (100 cm long × 50 cm wide × 50 cm deep). The containers were filled with clay (TN: 2.77 ± 0.54 mg.g^-1^, TP: 0.79 ± 0.19 mg.g^-1^) and to maintain a moist habitat, 1-cm-deep lake water (TN: 0.6 ± 0.2 mg. L^-1^; TP: 0.04 ± 0.01 mg. L^-1^) was maintained above the substrate surface throughout the pre-cultivated period. The plant materials were pre-cultivated in the greenhouse for approximately 2 months before the experiment was set up. On June 20, 2017, we selected 220 morphologically identical plants without shoots. Sixteen plants were randomly selected and dried to determine their initial biomass. The 204 remaining plants (height: approximately 25 cm; initial biomass: mean ± SE, 5.66 ± 0.58 g) were selected for the experiment.

### Experimental Design

**FIGURE 1:**
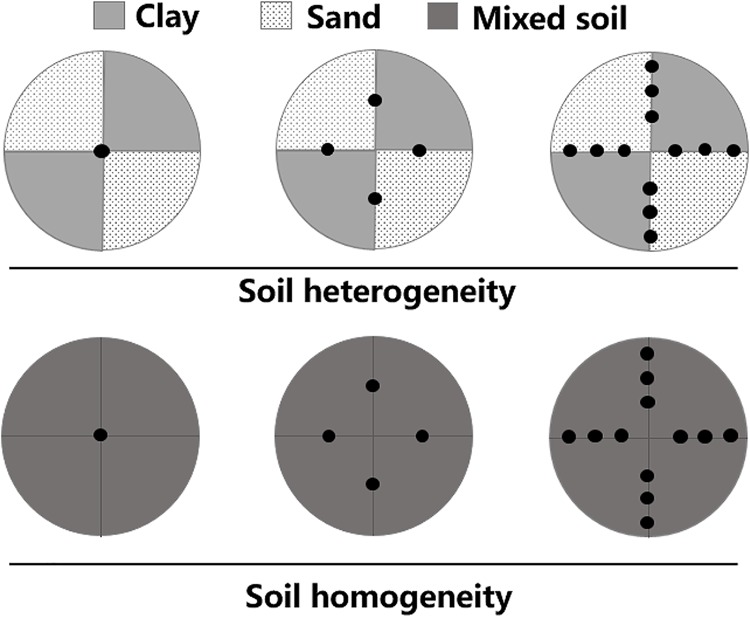
Schematic representation of the experimental design. Soil heterogeneity treatment design: light gray and lightly stippled areas represent high (clay) and low (sand) soil nutrient patches, respectively. Soil homogeneity treatment design: the area in dark gray was filled with an even mixture of the same volume of clay and sand. The total amounts of soil nutrients were thus the same in both treatments. Black dots represent where *M. aquaticum* was planted in three density-level treatments: one, four, or twelve plants per pot.

The experiments used a two-factorial design of plant density treatments and soil treatments. Plants were subjected to three density treatments (one, four, or twelve plants per container) and two soil treatments (homogeneous or heterogeneous; Figure 1). Each experimental container was 60 cm in height × 60 cm in diameter. There were six container replicates for each of the six treatments and thus 36 containers in total. The heterogeneous soil treatment was composed of two contrasting patches of equal volumes of clay (total nitrogen content: 2.98 ± 0.64 mg.g^-1^, total phosphorus content: 0.83 ± 0.21 mg.g^-1^, organic material content: 45.11 ± 2.67 mg.g^-1^, mean ± SE) and pure sand. For the homogeneous soil treatment, each container was a mixture of the same total amounts of clay and sand as in the heterogeneous treatments. The total amount of soil nutrients was the same in all treatments.

During the experiment, the experimental units were randomly repositioned every week to avoid the potential effects of environmental heterogeneity (such as light) and 1-cm-deep lake water (TN: 0.6 ± 0.2 mg. L^-1^; TP: 0.04 ± 0.01 mg. L^-1^) was maintained above the substrate surface. The diurnal variation in temperature, humidity and illumination was recorded each day by a hygrothermograph and an illuminometer in the greenhouse. The mean values of temperature, humidity, and illumination in the greenhouse were 27.58 ± 0.62°C, 64.52 ± 1.22% and 2892.06 ± 321.93 μmol. m^-2^.s^-1^ (mean ± SE), respectively.

After approximately 90 days of growth, on September 23, 2017, the total number of shoots was recorded, and the total length of shoots and plant height of each plant were measured. Each plant was then divided into aboveground (leaves and stem) and belowground (roots) parts, dried at 70°C for 72 h and weighed. The relative competition intensity (RCI) and log response ratio of biomass (LnRR) were calculated as follows:

$$\begin{array}{c} Relative competition intensity (RCI) =\frac{\left(B_{\text{mono}}-B_{\text{mix}}\right)}{Bmix}\\ Log response ratio of biomass (LnRR) = Ln\left(\frac{B_{\text{mono}}}{B_{\text{mix}}}\right) \end{array}$$

where *B_mono_* is the total biomass in the absence of competition (i.e., one-plant density treatment), and *B_mix_* is the average biomass of a plant in each container in the presence of competition (i.e., the four-plant and twelve-plant treatments) (Grace, 1995; Armas et al., 2004).

### Statistical Analyses

In this experiment, six experimental treatment groups were analyzed, with six samples in each treatment group. The total biomass, aboveground biomass and belowground biomass were transformed using the log_10_(x) function. Thus, all experimental data met the assumptions of normality and homogeneity of variance prior to analysis. Two-way ANOVAs were used to test the effects of plant density and soil treatments on the growth traits and intensity of intraspecific competition of *M. aquaticum*. Duncan’s test was used to examine the differences in trait values among the treatments. All data analyses were conducted using SPSS 22.0 (SPSS, Chicago, IL, United States).

## Results

### Effects of Soil Nutrient Heterogeneity and Density Treatments on the Growth of *M. aquaticum*

**FIGURE 2:**
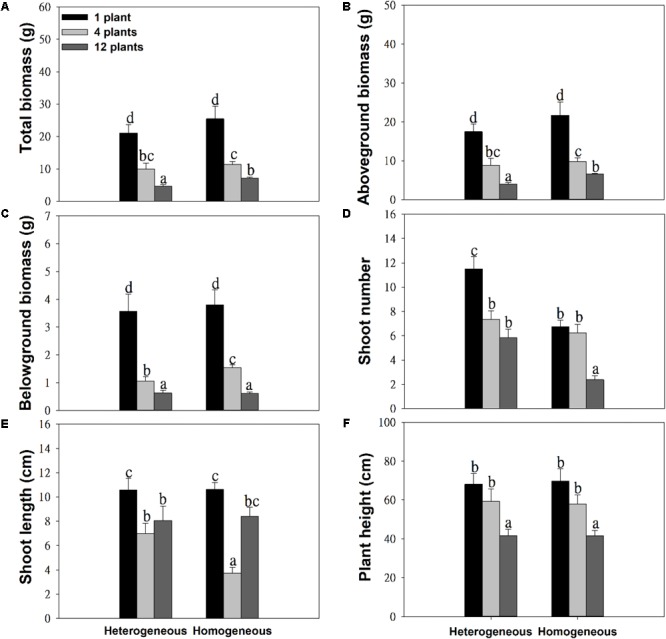
Effects of soil substrate type and plant density on **(A)** total biomass, **(B)** aboveground biomass, **(C)** belowground biomass, **(D)** shoot number, **(E)** shoot length, and **(F)** plant height of *M. aquaticum*. Values represent the mean ± SE. Means with different small letters are significantly different at *P* < 0.05 between the different treatments.

The soil substrate type had a significant effect on the total biomass, aboveground biomass and number of shoots (Table 1). However, it had no significant effects on belowground biomass, shoot length or plant height (Figure 2C,E,F and Table 1). For example, in the twelve-plant density treatment, soil nutrient heterogeneity significantly reduced total biomass by 38.94% and aboveground biomass by 35.46% but significantly increased the 1.46-fold number of shoots compared to plants grown in homogeneous soil (Figure 2A,B,D). Density treatments had a significant influence on the growth performance of *M. aquaticum*, and the growth traits of the low-density treatment were significantly greater than those of the high-density treatment (Figure 2A–F and Table 1). For example, soil nutrient heterogeneity significantly increased shoot number by 70.37% in single-plant treatments and shoot length by 87.17% in four-plant treatments compared to plants grown in homogeneous soil.

**Table 1:** *F*-value and significance of two-way ANOVA results for effects of soil substrate type (S) and plant density (D) on measures of biomass, morphological traits, and intraspecific competition of *M. aquaticum*.

	Substrate		Plant	
	type (S)		density (D)		S × D	
	*F*	*P*	*F*	*P*	*F*	*P*
Total biomass^a^ (g)	7.394	**0.010**	63.187	**<0.001**	1.076	0.352
Aboveground biomass^a^ (g)	7.844	**0.008**	55.947	**<0.001**	1.515	0.234
Belowground biomass^a^ (g)	2.410	0.129	96.868	**<0.001**	1.472	0.243
Number of shoots	33.340	**<0.001**	29.253	**<0.001**	4.007	**0.027**
Shoot length (cm)	2.266	0.141	22.618	**<0.001**	3.295	**0.049**
Plant height (cm)	0.001	0.982	14.272	**<0.001**	0.038	0.963
LnRR	1.554	0.225	27.758	**<0.001**	1.754	0.198
RCI	0.162	0.691	20.750	**<0.001**	0.910	0.350

*^∗^Significant *P*-values are shown in bold. ^∗^That little “a” shown that data were transformed using the log_*10*_(x) function.*

Except for the number and length of shoots, the effect of density treatments on the growth performance of *M. aquaticum* showed the same trends in the soil heterogeneity or homogeneity treatments (Figure 2A–F and Table 1). The interaction effect of soil and density treatment had significant effects on the shoot number and shoot length of *M. aquaticum* (Table 1).

### Effects of Soil Nutrient Heterogeneity Treatments on the Intraspecific Competition Intensity of *M. aquaticum*

**FIGURE 3:**
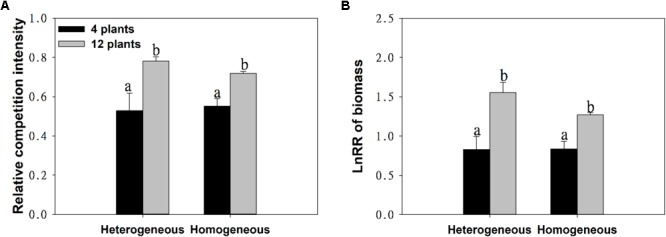
Effects of soil substrate type and plant density on **(A)** relative competition intensity, **(B)** LnRR of biomass of *M. aquaticum*. Values represent the mean ± SE. Means with the different small letters are significantly different at *P* < 0.05 between the different treatments.

With the increase in plant density, the intraspecific competition intensity increased gradually. For example, the log response ratio of biomass (LnRR) significantly increased, by approximately 51.73% ∼ 87.38%, both when soil nutrients were homogeneous and when they were heterogeneous (Figure 3A,B and Table 1). These results show that the intraspecific competition of *M. aquaticum* gradually intensified with increasing plant density. Although the intraspecific competition intensity of the high-density treatment was very high, soil nutrient heterogeneity did not aggravate intraspecific competition in *M. aquaticum* (Figure 3A,B and Table 1).

## Discussion

### Soil Nutrient Heterogeneity May Be a Promoter of Invasion and Spread in *M. aquaticum*

Heterogeneous soil had no effects on most growth traits except shoot numbers in the one-plant density treatment (Figure 2). In addition, plants accumulated less biomass when soil nutrient availability was heterogeneous at high density (Figure 2). Few studies have found that clonal plants grew more biomass in the heterogeneous than in the homogeneous soil treatment (Hutchings and Wijesinghe, 2008; Zhou et al., 2012; You et al., 2014). However, the positive effect of soil nutrient heterogeneity on the growth performance of plants may gradually decrease because the soil nutrients become limited in high-density populations (Day et al., 2003a; Dong et al., 2015). For example, soil nutrient heterogeneity has a short-term effect on the growth cycle of *Cardamine hirsuta*, and it does not have a long-term impact (Day et al., 2003a,b). Furthermore, the long-term response of *Holcus lanatus* was to produce far less shoot biomass in the heterogeneous treatment than expected under the homogeneity treatment (Fransen and Kroon, 2001). In addition, the growth performance of clonal plants in heterogeneous soil conditions may be correlated with the spatial scale of heterogeneity (Wijesinghe and Hutchings, 1997; Wang et al., 2016). For example, the growth of *Glechoma hederacea* was dependent on the spatial scale of soil nutrient heterogeneity (Wijesinghe and Hutchings, 1997), but *Alternanthera philoxeroides* displayed generally similar, scale-independent performance in most traits under different scales of soil nutrient heterogeneity (Wang et al., 2016). We speculate that the benefits of environmental heterogeneity to clonal plants may be correlated with spatial or temporal scale.

However, our study found that *M. aquaticum* produced significantly more and longer shoots in the heterogeneous soil than in the homogenous soil treatment. Morphological plasticity enables the adaptation of clonal plants to heterogeneous environments, which probably benefits clonal plants through the optimization of plant performance (Kroon and Hutchings, 1995). Clonal integration may help *M. aquaticum* adapt to the heterogeneous distribution of resources. For example, clonal integration can boost the growth of *M. aquaticum* when subjected to heterogeneity in resource supply in changing environments (You et al., 2013). Clonal plants can share photosynthates, mineral nutrients, or water among individual subunits through clonal integration, which increases the survival of clonal plants when they experience heterogeneous distribution of resources (Xiao et al., 2006; Yu et al., 2019). Thus, we predict that the positive response of clonal plants to environmental heterogeneity may be correlated with clonal integration and morphological plasticity.

Except for a higher number of shoots, slight effects and even lower biomass of *M. aquaticum* were found in heterogeneous nutrient treatments. This result is not completely in agreement with the prediction of our hypothesis but is consistent with previous studies, for example, nutrient heterogeneity does not affect the growth of a species of the same genera, *Myriophyllum spicatum* (Li et al., 2016). *Vallisneria natans* and *Prosopis glandulosa* did not show any significant changes in growth performance under spatially heterogeneous conditions (Maestre and Reynolds, 2007; Xie et al., 2007). On the other hand, *M. aquaticum* survival and spread depends solely on vegetative reproduction via fragmentation (Sutton, 1985). The higher number of shoots may aid in the spread of this species. Thus, soil nutrient heterogeneity can have a positive effect on invasive success in *M. aquaticum*, especially through occupying space and spreading.

### Soil Nutrient Heterogeneity Could Not Change the Competitive Relationship Among Individuals in the Population of *M. aquaticum*

Spatial heterogeneity in soil nutrient availability can influence interspecific or intraspecific competition (Day et al., 2003b; Van et al., 2011; Mommer et al., 2012; Zhou et al., 2012). Plants can proliferate roots and ramets in nutrient-rich substrate patches to improve nutrient absorption efficiency in heterogeneous environments (Fransen et al., 2001; Day et al., 2003b; Dong et al., 2015). This may lead to fierce competition between plant species in heterogeneous conditions because the foraging organs of neighboring plants would be concentrated in a smaller patch of the soil. A previous study showed that soil nutrient heterogeneity, acting through its effect on competition, is likely to be an important influence on community structure and composition (Day et al., 2003b).

However, in this study, we found that soil nutrient heterogeneity could not alter the intraspecific competition of *M. aquaticum*. This was not due to an absence of competition, as plants grew less at high than at low density treatments both when soil nutrients were homogeneous and when they were heterogeneous. The reasons for these results may be as follows: First, clonal plants can share resources among individual subunits by physiological integration (Hutchings and Wijesinghe, 1997; Hellstrom et al., 2006), which may alleviate the severe competition between individual subunits in nutrient- patches (Novoplansky, 2009; Dong et al., 2015). Second, resource heterogeneity can significantly affect plant competition when individuals are not genetically identical (Day et al., 2003b; Zhou et al., 2012) or may be due to the differences in plant ability to place foraging organs in areas where available resources are high (Wijesinghe et al., 2001; Bliss et al., 2002).

In heterogeneous environments, local adaptation to particular environmental conditions may also occur within plant populations on a much smaller geographical scale (Hangelbroek et al., 2004). Substrate characteristics can determine macrophyte community structure within lakes from a combination of both regional-scale multi-lake studies and smaller-scale studies (Johnson and Ostrofsky, 2004). Thus, our study showed that soil nutrient heterogeneity, especially for fine-scale heterogeneity, does not increase competition between individuals of *M. aquaticum*.

## Conclusion

We conclude that spatial heterogeneity in soil nutrient availability is likely to be a primary promoter of invasive success in *M. aquaticum*. In the case of *M. aquaticum*, the positive effects of soil nutrient heterogeneity are shown mainly in the morphological characteristics of individual clonal shoots and cannot change the competitive relationship of clonal plants such as *M. aquaticum*. Therefore, spatial heterogeneity in soil nutrient availability may have a positive effect on the invasive spread of *M. aquaticum*. In the future, a more intimate knowledge of how diversified environmental heterogeneity due to various ecological factors at different scales affects the invasive performance of alien species is needed. This will enable a better understanding of the dynamic changes in invasive species composition and richness in aquatic ecosystems.

## Data Availability

The datasets generated for this study are available on request to the corresponding author.

## Author Contributions

CL and DY designed the experiment and edited the manuscript text. HY and NS performed the experiment. NS and SY wrote the manuscript text and executed statistical analysis.

## Conflict of Interest Statement

The authors declare that the research was conducted in the absence of any commercial or financial relationships that could be construed as a potential conflict of interest.
